# Outcome of Percutaneous Release of Tennis Elbow: A Non-Randomized Controlled Trial Study

**DOI:** 10.7759/cureus.952

**Published:** 2017-01-02

**Authors:** Sagar Panthi, Kishor Khatri, Krishna Kharel, Subin Byanjankar, Rahul Shrestha, Jay R Sharma, Raju Vaishya, Amit kumar Agarwal, Vipul Vijay

**Affiliations:** 1 Orthopaedics and Trauma Surgeon, Nepalgunj Medical College and Teaching Hospital; 2 Orthopaedics and Trauma Surgeon, Lumbini Zonal Hospital; 3 Orthopaedics, Tilottama Hospital; 4 Ortthopaedics, Lumbini Medical College, Nepal; 5 Orthopaedics, Lumbini Medical College, Nepal; 6 Orthoapedics, Anandaban Hospital, Lalitpur; 7 Orthopaedics, Indraprastha Apollo Hospitals

**Keywords:** percutaneous tenotomy, tennis elbow, 18 g hypodermic needle

## Abstract

**Background:**

Tennis elbow is a common disorder of the upper extremity. It can be treated conservatively in the majority of patients, but some resistant cases eventually can be treated by percutaneous release with good functional outcome.

**Materials and methods:**

This non-randomized control trial was conducted at the Department of Orthopaedics Surgery in a tertiary care hospital from July 2015 to June 2016 on 50 patients who underwent percutaneous release of the common extensor origin using an 18 gauge hypodermic needle. These patients did not respond to conservative treatment including rest, nonsteroidal anti-inflammatory drugs (NSAIDS) and local steroid injections. The outcome was graded as Excellent, Good, Fair, and Poor.

**Results:**

Fifty patients (50 elbows) were included in the study. Thirty-two patients were female (64%), and 18 were male (36%). The right side was affected in 37 patients (74%) and left side in 13 (26%). The time taken to achieve a completely pain-free elbow ranged from one day to two months (average of 26.2 days). Those who did not achieve a pain-free elbow had a residual pain of 1.5 to six on the visual analogue scale (VAS) (average 2.32). Excellent outcome was noticed in 24 patients (48%); Good result in eight patients (36% ); Fair in four patients (eight percent) and Poor in four patients (eight percent).

**Conclusion:**

Tennis elbow probably results from the degenerative tear of the common extensor origin, and a percutaneous tenotomy using an 18 gauge hypodermic needle is a simple, safe, patient-friendly, efficient, and easily reproducible method of treating tennis elbow in those who are resistant to conservative treatment, and it can be done as an outpatient procedure.

## Introduction

Lateral epicondylitis or ‘tennis elbow' is a common and well-known condition that causes pain on the lateral aspect of the elbow. It was first described in 1873 by Runge. The incidence of tennis elbow in the population varies from one to three percent [1]. It is not exclusively secondary due to the playing of tennis and is commonly seen in people who do heavy manual work [2].

Many theories have been suggested to explain the etiology of this condition like bursitis, periostitis, infection, aseptic necrosis, neuritis of branches of the radial nerve, radiohumeral synovitis, and irritation of the collateral ligament. The most widely held theory is that there are macroscopic or microscopic tears in the common extensor origin as described by Cyriax and others [3].

Symptoms may include local tenderness over the lateral epicondyle, pain in the extensor muscles induced by gripping or resisted extension movements of the wrist [4]. On examination, pain may be exacerbated by resisted wrist extension in the pronated position. It is worse with the elbow at full extension. The range of motion of the wrist and elbow is usually complete [5].

Greater than 90% of these patients can be successfully treated nonoperatively [6]. Those who do not respond to conservative treatment are usually offered surgery. A variety of surgical procedures for treating tennis elbow has been described in the literature [7-8]. One of them is tenotomy of the common extensor origin at the elbow. Many authors have now published their results of releasing the common extensor origin percutaneously using either the surgical blade or the hypodermic needle under general anesthesia [9-11]. It is a simple operation with minimal morbidity and good-to-excellent results in most of the studies. We present our results of percutaneous tenotomy of the common extensor origin conducted in the outpatient department using the bevel of an 18 gauge hypodermic needle for the tenotomy instead of a surgical blade. Informed consent was obtained from the patients for this study.

## Materials and methods

Our non-randomised control trial study was conducted at the Department of Orthopaedics Surgery in a tertiary care hospital from July 2015 to June 2016 on 50 patients who underwent percutaneous release of the common extensor origin using an 18 gauge hypodermic needle (Table 1).

**Table 1:** Table showing inclusion and exclusion criteria

Inclusion Criteria	Exclusion criteria
Age more than 30 years and less than 60 years	Age less than 30 years and more than 60 years
Pain for six months duration not responding to medical treatment and one dose of steroid injection	Acute pain
	Calcification on lateral epicondyle on X-ray

Data was collected by going through the patient's medical records, and a follow-up questionnaire was asked to assess the outcome and patient satisfaction with the procedure. The diagnosis of tennis elbow was made on the consistent signs of tenderness directly over the lateral epicondyle, pain over the lateral epicondyle on an extension of the wrist against resistance and “handshake sign,” where the patient with tennis elbow experiences pain in the lateral epicondyle on the handshake. Fifty patients with age more than 30 years and less than 60 years with the duration of pain for more than a six-month period not responding to medical therapy and one dose of local steroid injection were included in our study. Fifty patients or 50 elbows were included in the study. The age of the patients ranged from 35 to 51 years (average 42.2 years). The pain duration before the surgery ranged from three months to three years (average 9.3 months).

**Figure 1:**
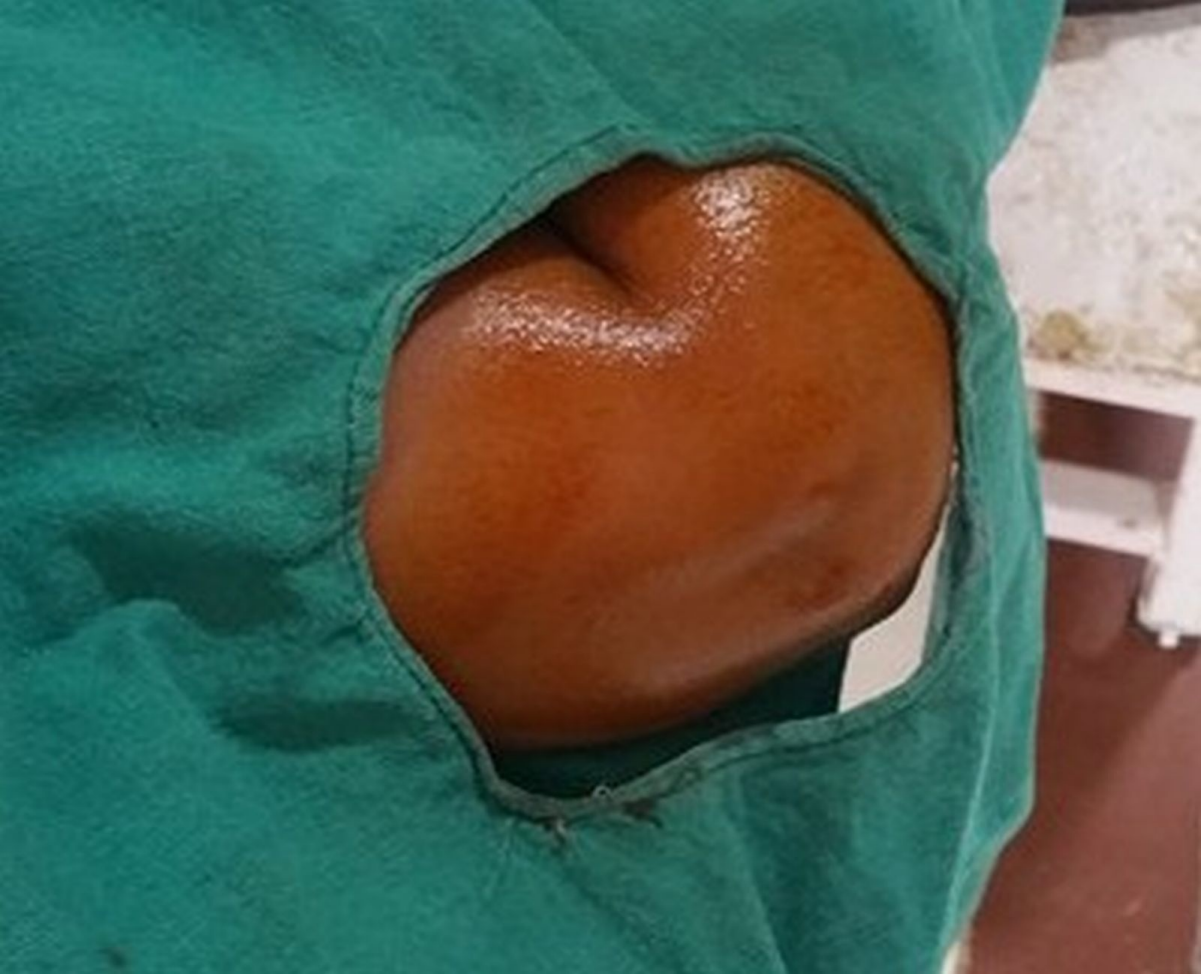
Image showing painting and draping of the lateral epicondyle

**Figure 2:**
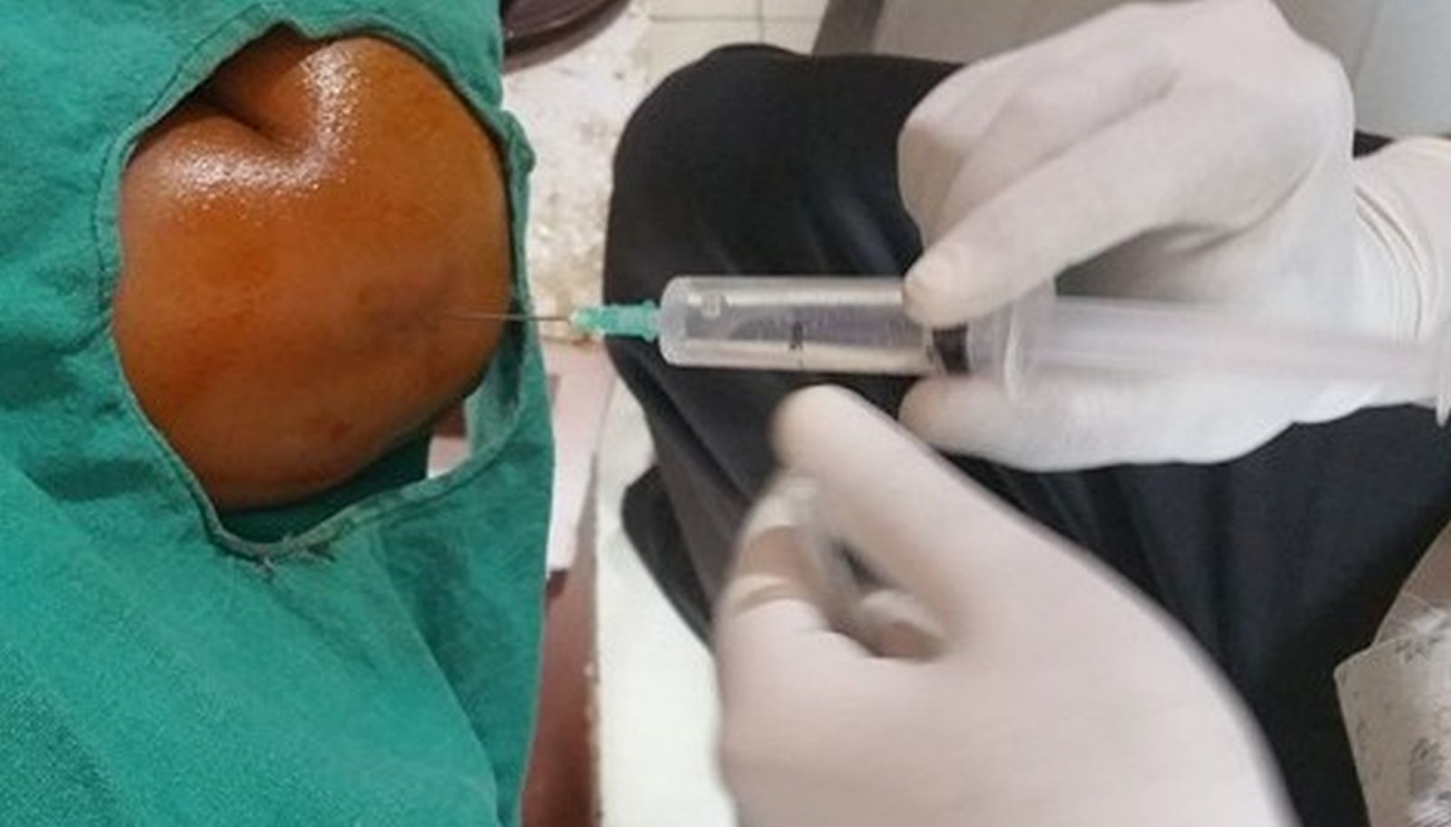
10 ml of two percent Xylocaine inserted over the lateral epicondyle at maximum tenderness

All the procedures were performed by the author in the outpatient minor procedure room. The technique for the procedure is described below:

1. With the patient seated comfortably on a chair and the forearm resting passively on an examination couch by the side, the elbow was flexed to 90 degrees and the wrist passively flexed to around 60 degrees.

2. After preparing the entire aspect of the lateral elbow with Betadine solution, 10 ml of two percent lignocaine (local anesthetic) was infiltrated by a 30 G needle around the entire common extensor origin (Figures 1-2).

 

**Figure 3:**
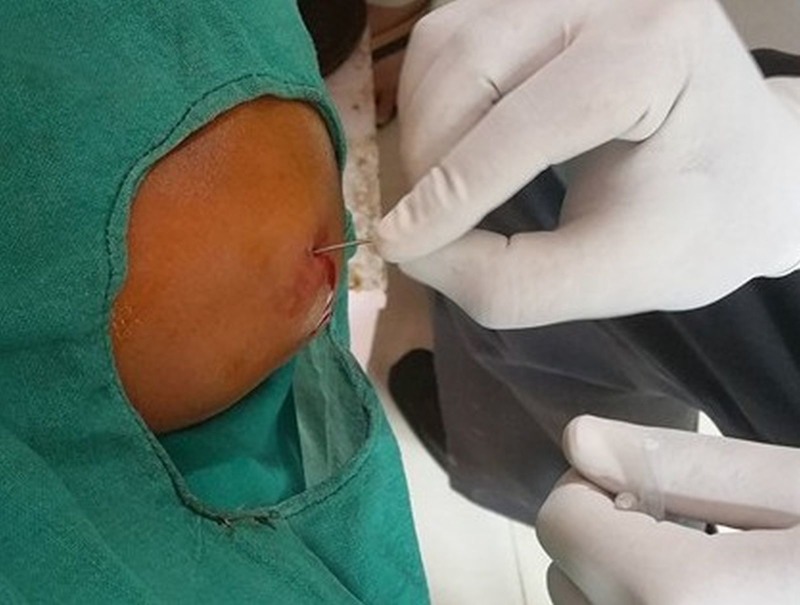
Percutaneous tenotomy with an 18 G hypodermic needle

3. After the local anesthetic had taken effect, an 18 G needle was introduced through the skin, and the bevel of the needle was used to divide the extensor origin at the site of maximum tenderness. The radial nerve was protected by staying within the extensor origin (Figure 3).

4. The needle puncture site was sealed using a Band-Aid, and a tennis elbow brace was applied. Postoperatively, 1 g of paracetamol tablet was given four times a day for few days. The tennis elbow brace was discarded after the pain resolved, and normal activity of the limb was resumed as quickly as possible.

Patient outcome and satisfaction were graded as excellent, good, fair, and poor according to pain relief (VAS score) and function.

## Results

Out of 50 patients, 32 were female (64%), and 18 were male (36%). All patients had unilateral tennis elbow. The right side was involved in 37 patients (74%) and the left side in 13 patients (26%) (Table 2).

**Table 2:** Table showing side involved (n=50)

SIDE INVOLVED	NO. OF ELBOWS	PERCENTAGE
Right	37	74%
Left	13	26%

The age range of patients was 35 to 51 years. The outcome results were graded as excellent, good, fair, and poor as shown in Table 3.

**Table 3:** Table showing outcome grading based on the pain score

GRADING	PARAMETER
Excellent	Full return to all activity with no pain.
Good	Full return to all activity with occasional mild pain.
Fair	Pain with normal activities; significant pain with heavy activities.
Poor	Little or no relief of preoperative symptoms.

Forty-six patients (96%) were satisfied with the results of the percutaneous release. In four patients, the pain didn’t subside and was managed with the surgical release and the pain subsided later. Four patients in whom the symptoms did not subside were farmers by profession and the cause of pain may be due to their immediate return to strenuous activity. Twenty-four elbows (48%) had an excellent outcome, 18 (36%) had good, four (eight percent) had satisfactory, and four (eight percent) had poor outcomes as shown in Table 4.

**Table 4:** Table showing outcome of procedure (n=50)

GRADING	NUMBER OF PATIENTS	PERCENTAGE
Excellent	24	48%
Good	18	36%
Fair	4	8%
Poor	4	8%

Forty-eight percent of the patients had the excellent or good outcome. Forty of the 50 elbows (80%) became completely pain-free in one day to two months (average of 26.2 days). Those who did not achieve a pain-free elbow had a residual pain of 1.5 to six on the VAS scale (average 2.32).

## Discussion

Greater than 90% of tennis elbow patients can be successfully treated conservatively by rest, activity modification, analgesics, and local steroid injection. Since different etiologies have been proposed for this condition, a variety of surgical options have been tried depending upon the etiology. These include:

1. open/percutaneous division of the common extensor origin,

2. excision of pathological tissue at the extensor carpi radialis brevis (ECRB), 

3. repair of the longitudinal defects,

4. denervation of the lateral epicondyle both by isolation of the individual nerve branches (all of the radial nerve),

5. decompression of the radial nerve as it dives deep to the proximal border of the superficial head of the supinator muscle and Arcade of Frohse,

6. division of the annular ligament,

7. excision of intraarticular synovial folds [7],

8. and surgical lengthening of the ECRB tendon.

The results of the percutaneous release of the common extensor origin have been very attractive regarding simplicity, safety, minimal morbidity to the patients, and good to excellent outcome in the majority of patients.

Grundberg and Dobson reported 29 of 32 operated cases having excellent or good results, but they have not mentioned any criteria for the same in their publication. Similarly, Yerger and Turner operated on 149 patients with more than 90% achieving excellent or good results. Once again, they have not mentioned any criteria for the same in their publication. Baumgard and Schwartz delivered excellent results in 32 of 35 patients they operated. Their results were termed Excellent (no preoperative symptoms), Good (improvement of preoperative symptoms) or Poor (no improvement of preoperative symptoms) depending on the outcome symptoms. Since our outcome criteria are different from the one mentioned in literature and because two of the other publications do not have any outcome criteria for excellent or good results at all, the result of our study with 80% good or excellent results cannot be compared with that of others.

All of these chronic tennis elbow patients had undergone various modalities of non-operative treatments including multiple steroid injections for the condition before being undertaken for surgery. It is hard to believe that they do not affect microscopic changes in some way at the local site. No published studies have examined specimens from patients with an acute diagnosis of lateral tennis elbow syndrome. After going through the literature, we tend to agree with those who believe that it results from a gradual degenerative tear of the common extensor origin [5]. We believe that tenotomy of the common extensor tendons and scraping of the epicondylar region using the beveled end of an 18 G needle expedites the healing process of the degenerative tendon by converting a chronic inflammatory condition to an acute inflammatory condition which heals rapidly, thereby relieving the pain of tennis elbow which is not amenable to conservative treatment.

As this is a non-randomised control trial there is a possibility of observer bias, which can be avoided by doing a randomized control trial. Another limitation of our study could be the halo effect, which can be avoided by increasing the number of observers.

## Conclusions

Most patients with lateral epicondylitis respond to conservative treatment. In resistant cases in which surgical treatment appears necessary, the percutaneous release of the common extensor origin may be considered as a first choice and can be done as an outpatient procedure.
